# Informative gene network for chemotherapy-induced peripheral neuropathy

**DOI:** 10.1186/s13040-015-0058-0

**Published:** 2015-08-12

**Authors:** Cielito C. Reyes-Gibby, Jian Wang, Sai-Ching J. Yeung, Sanjay Shete

**Affiliations:** 1 Department of Emergency Medicine, The University of Texas MD Anderson Cancer Center, Houston, TX 77030 USA; 2 Department of Biostatistics, The University of Texas MD Anderson Cancer Center, Houston, TX 77030 USA; 3 Department of Epidemiology, The University of Texas MD Anderson Cancer Center, Houston, TX 77030 USA

## Abstract

**Background:**

Host genetic variability has been implicated in chemotherapy-induced peripheral neuropathy (CIPN). A dose-limiting toxicity for chemotherapy agents, CIPN is also a debilitating condition that may progress to chronic neuropathic pain. We utilized a bioinformatics approach, which captures the complexity of intracellular and intercellular interactions, to identify genes for CIPN.

**Methods:**

Using genes pooled from the literature as a starting point, we used Ingenuity Pathway Analysis (IPA) to generate gene networks for CIPN.

**Results:**

We performed IPA core analysis for genes associated with platinum-, taxane- and platinum-taxane–induced neuropathy. We found that *IL*6, *TNF*, *CXCL*8, *IL*1*B* and *ERK*1/2 were the top genes in terms of the number of connections in platinum-induced neuropathy and *TP*53, *MYC*, *PARP*1, *P*38 *MAPK* and *TNF* for combined taxane-platinum–induced neuropathy.

**Conclusion:**

Neurotoxicity is common in cancer patients treated with platinum compounds and anti-microtubule agents and CIPN is one of the debilitating sequela. The bioinformatic approach helped identify genes associated with CIPN in cancer patients.

## Introduction

Chemotherapy-induced peripheral neuropathy (CIPN) is a debilitating condition. CIPN is a dose-limiting toxicity for chemotherapy agents, such as oxaliplatin, cisplatin, and platinum [1–4]. Chemotherapeutic agents may cause structural damage to peripheral nerves, which can result in aberrant somatosensory processing by the peripheral and/or central nervous system. The symptoms of CIPN vary depending on the type of chemotherapy administered and which nerve fibers are affected. Unusual sensations (paresthesia), numbness, balance problems or pain may result from chemotherapies that affect the sensory nerve fibers. When motor nerves are affected, patients may report weakness of the muscles in the feet and hands.

Patients who suffer from CIPN have a higher risk (as much as threefold higher) of developing neuropathic pain (NP) [5]. Defined as “pain initiated or caused by primary lesion or dysfunction in the nervous system,” NP occurs in nearly 40 % of patients who experience cancer pain [6, 7]. Patients with NP experience higher pain intensity and less effective control of their pain with conventional analgesia [8]. Further, patients with NP rate their level of pain relief to be significantly lower than those with nociceptive pain (defined as pain caused by activation of primary afferents in somatic or visceral tissues) in response to a single dose of an opioid [8, 9]. Patients with NP report twice as many visits to their health care provider (*p* = 0.02) and take more prescription (50 % versus 19 %; *p* = 0.001) and over-the-counter medications (62.5 % versus 45 %; *p* = 0.08) for pain than those without NP [5].

Published guidelines for the initial treatment of NP include the use of gabapentin, pregabalin, carbamazepine, tricyclic antidepressants, oxycodone, morphine, methadone, tramadol, duloxetine, and venlafaxine [10, 11]. However, placebo-controlled trials have shown that medications such as gabapentin [12] and glutamine [13] have no statistically significant effects on NP. Animal and human studies have been conducted to identify the best ways to treat and manage NP [14–20]. Because CIPN is a risk factor for the development of NP in cancer patients, a better understanding of the potential biological mechanisms underlying CIPN has huge clinical significance.

Host genetic variability has been implicated in many pain conditions, including neuropathy. Each of these studies assessed different therapeutic agents and different genetic mechanisms. However, it is understood that as a complex trait, several genes are implicated in CIPN. Bioinformatics provides tools for using large-scale information to produce comprehensive networks of genes and the underlying biological pathways implicated in a phenotype. Therefore, in this study, we used the Ingenuity Pathway Analysis (IPA), a bioinformatic tool for analyzing biological data, and performed a comprehensive network-based approach to identify genes implicated in neuropathy induced by chemotherapy agents. Compared to traditional regression approaches, network-based approaches can provide a holistic picture that captures the complexity of intracellular and intercellular interactions in diseases [21]. Furthermore, the network-based approaches can identify genes and pathways related to a disease or phenotype, which will lead to a better understanding of the underlying biological mechanisms [22]. Further, networks generated from IPA core analysis may suggest new candidate genes for future studies of CIPN.

## Methods

With the goal of identifying a comprehensive list of genes and potentially novel genes associated with CIPN, we first conducted a literature search as described below. Using genes pooled from the literature as a starting point, we used IPA to generate gene networks for CIPN.

### Literature review

Using the PubMed database, we performed a comprehensive literature review, limiting our search to human studies and articles published in English before July 2014. The primary purpose of the literature search was to identify genes associated with CIPN in cancer patients. The terms we used were “cancer neuropathy SNP,” “cancer neuropathy SNPs,” “cancer neuropathy gene,” “cancer neuropathy genes,” “cancer neurotoxicity SNP,” “cancer neurotoxicity SNPs,” “cancer neurotoxicity gene” and “cancer neurotoxicity genes.” We then screened the resulting articles based on the title, abstract, and the full text, and excluded duplicate articles. Next, we manually searched the reference lists of the articles identified in our initial search and those in related review articles to identify additional relevant articles (Table 1). From these studies, we retrieved the information about genes harboring or close to the significantly associated genetic variants (SNPs or haplotypes) and included those genes in the IPA. In particular, we included only those genes for IPA analysis that (1) have been replicated in an independent study or meta-analysis, (2) have at least one SNP that reached the genome-wide significance level, or (3) have a known biological functional significance (e.g., multi-drug resistance, drug metabolism, and mediating developmental events in the nervous system). We also summarized the information based on the different chemotherapy agents used for cancer patients.

**Table 1:** Number of articles obtained using different search terms

Search terms	# of articles by PubMed search	# of articles by initial screen	# of articles from references	# of articles included
cancer neuropathy SNPs(SNP)	30	20	36	56
cancer neuropathy genes(gene)	266	1	0	1
cancer neurotoxicity SNPs(SNP)	37	6	0	6
cancer neurotoxicity genes(gene)	349	1	0	1
Total	682	28	36	64

### Ingenuity pathway analysis

IPA (Ingenuity^®^ Systems, www.ingenuity.com) is a software that connects a list of molecules in a set of networks based on the scientific information contained in the Ingenuity Knowledge Base of biological interactions and functional annotations from millions of relationships between proteins, genes, complexes, cells, tissues, drugs, and diseases [23, 21]. In the networks, nodes are used to represent molecules (e.g., genes, chemicals, protein families, complexes, microRNA species and biological processes) [24] and lines connecting two molecules are used to represent the relationship between them. Many different types of relationships are considered in the IPA analyses, including activation, binding, causation, chemical-chemical interaction, expression enzyme catalysis, inhibition, biochemical modification, protein-protein binding and transcription.

In this study, we utilized the IPA core analysis function to generate relevant networks that identify additional genes that interact with the genes identified from the literature review (denoted as focus genes in IPA). The IPA core analysis function is a process to create networks on the basis of the focus genes [25]. The working hypothesis for network generation is that the biological function involves locally dense interactions; thus, IPA uses an algorithm to attempt to generate networks that are as densely connected as possible [26]. The network generation process first ranks the focus genes in decreasing order on the basis of triangular connectivity, which measures the number of triangular connections in which a gene functions (or pairs of genes to which a gene is connected). The most connected focus gene (the top ranked gene) is considered to be the starting seed gene. Next, the remaining focus genes that are in the neighborhood of the starting seed gene are added to generate the first seed gene network. A neighborhood is defined as a gene plus the genes exactly one connection away from that gene. Then the second seed gene network is identified from the focus genes that are not included in the first seed gene network. The process continues until all focus genes are represented in a relevant network. Subsequently, all smaller networks are combined to make larger networks by connecting seed gene networks through an additional non-focus gene. If the gene network does not reach the maximum network size (140 genes in this study), IPA will then connect additional genes/networks from its database to any of the genes involved in the gene network. Specifically, given a network, to identify additional genes to be added, IPA gives priority to the genes that have the largest overlap with the existing network and have the least number of neighbors. This property is measured using a metric called specific connectivity, which is calculated by dividing the number of genes in the intersection of the neighborhood and the existing network by the union of the number of genes in the neighborhood and the existing network. The gene with the highest specific connectivity score is included in the existing network. Importantly, the IPA analysis can exclude a focus gene from the resulting network if such a gene is less likely to have connections (i.e., biological relationships) with the network.

The resulting functions/pathways/networks are evaluated using the right-tailed Fisher’s exact test, which provides *p* values based on the null hypothesis that the association between a set of focus genes and a given function/pathway/network is due to random chance [25]. Specifically, if the final network includes *n* genes and *n*_*f*_ of them are focus genes, the *p* value is the probability of finding *n*_*f*_ or more focus genes in a set of *n* genes randomly selected from the IPA pre-specified database [26]. A score, which is assessed as -log_10_(p value), is used to rank the resulting functions/pathways/networks. We used a significance level of <10^−5^ in our study (score > 5) when selecting networks [21].

We limited the IPA analysis to human studies. In the IPA core analysis, we used the Ingenuity Knowledge Base as the reference set. In order to generate networks in the core analysis, we used the settings of a maximum of 140 genes per network and 25 networks per analysis, because the networks for up to 140 genes allow for the possibility that the same network can include all focus genes [27]. We reported the most interconnected genes in the networks as the key genes of interest, because highly connected molecules (called hubs) are typically associated with biological functions or diseases [22, 24, 21, 26, 27].

## Results

### Literature review

From our search of the PubMed database, we initially identified 682 articles. After screening the title, abstract and full text, we excluded 654 articles for the following reasons (Table 1): (1) not human studies; (2) not published in English; (3) meta-analysis study, review or letter to the editor; (4) clinical trial studies; (5) not genetic association studies; (6) not neuropathy-related phenotypes studies; (7) not cancer studies; and (8) duplicate articles from different searches. We then manually searched the reference lists from the resulting 28 articles and from related review articles about genetic neuropathy studies, and identified 36 more articles. As a result, we had a total of 64 articles from which we extracted information to identify the focus genes and perform the analyses through IPA.

Table 2 lists the information we retrieved from each of the studies, including the year of publication, first author, ethnicity of patient population, cancer type, sample size, phenotypes, and significant genes. These studies included different cancer sites and patients of different ethnic groups. Neuropathy (or neurotoxicity) in cancer patients is usually induced by the chemotherapy agents used in cancer treatment, such as oxaliplatin, cisplatin, and platinum, and is usually measured according to the National Cancer Institute’s Common Terminology Criteria for Adverse Events or Common Toxicity Criteria.

**Table 2:** List of genetic association studies for chemotherapy-induced neuropathy in cancer patients, sorted by publication year and name of first author

Year	First author	Ethnicity	Cancer type	Sample size	Phenotype	Significant genes
2003	Aplenc R [41]	W, AA, H	Acute lymphoblastic leukemia	533	Peripheral neuropathy	CYP3A4, CYP3A5
2004	Isla D [42]	W	Lung	62	Docetaxel-cisplatin-treated neurological	None
2006	Lecomte T [43]	W	Gastrointestinal solid tumors	64	Oxaliplatin-related cumulative neuropathy	GSTP1
2006	Sissung TM [44]	W	N/A	26	Paclitaxel-induced neuropathy	ABCB1
2007	Gamelin L [45]	W	Colon, rectum	145	Oxaliplatin-induced neurotoxicity	AGXT
2007	Marsh S [46]	N/A	Ovarian	914	Paclitaxel/docetaxel-induced neuropathy	None
2007	Oldenburg J [47]	W	Testicular	238	Self-reported chemotherapy-induced long-term toxicities	GSTP1
2007	Ruzzo A [48]	W	Colorectal	166	Oxaliplatin-induced neurotoxicity	GSTP1
2008	Keam B [49]	A	Gastric	73	Peripheral sensory neuropathy	None
2008	Pare L [50]	W	Colorectal	126	Cumulative oxaliplatin-induced neuropathy	None
2008	Sissung TM [51]	N/A	Prostate	73	Docetaxel-induced neuropathy	ABCB1
2009	Argyriou AA [52]	W	Colorectal	62	Oxaliplatin-induced peripheral neuropathy	None
2009	Goekkurt E [53]	W	Gastric	134	Neurotoxicity	GSTP1
2009	Green H [54]	W	Ovarian	38	Sensory/motor neuropathy	None
2009	Kim HS [55]	A	Epithelial ovarian	118	Taxane/platinum- induced neurotoxicity	ERCC1
2009	Kweekel DM [56]	W	Colorectal	91	Neurotoxicity	None
2009	Mir O [57]	W	Breast, lung, prostate	58	Docetaxel(Taxotere)-induced peripheral neuropathy	GSTP1
2009	Seo BG [58]	A	Gastric	94	Neuropathy	None
2010	Antonacopoulou AG [59]	W	Colorectal	55	Chronic oxaliplatin-induced peripheral neuropathy	ITGB3
2010	Boige V [60]	W	Colorectal	349	FOLFOX-induced severe neurologic toxicity	None
2010	Chen YC [61]	A	Colorectal	166	Oxaliplatin-induced chronic cumulative neuropathy	GSTP1
2010	Cho HJ [62]	A	Diffuse large B-cell lymphoma	94	Chemotherapy-related neurotoxicity	None
2010	Inada M [63]	A	Colorectal	51	Oxaliplatin-induced peripheral neuropathy	ERCC1, GSTP1
2010	Kanai M [64]	A	Colorectal	82	Early-onset oxaliplatin-induced neuropathy	None
2010	Khrunin AV [65]	W	Ovarian	104	Cisplatin-based neuropathy	GSTM1, GSTM3
2010	Li QF [66]	A	Gastric	92	Neurological toxicity	GSTP1
2010	McLeod HL [67]	W, A, AA, H	Metastatic colorectal	520	Diarrhea, vomiting, paresthesia, febrile neutropenia and neutropenia	GSTP1
2010	Ofverholm A [68]	W	Breast, ovarian	36	Occurrence and degree of neurotoxicity	None
2010	Rizzo R [69]	W	Breast	95	Taxane-induced hypersensitivity and sensory neuropathy	None
2011	Basso M [70]	W	Colorectal, pancreatic, bile ducts	40	Acute oxaliplatin neurotoxicity	SK3
2011	Bergmann TK [71]	W	Ovarian	119	Sensory neuropathy	None
2011	Bergmann TK [72]	W	Ovarian	92	Sensory neuropathy	None
2011	Broyl A [73]	W	Multiple myeloma	369	Bortezomib/vincristine-induced peripheral neuropathy	RHOBTB2, CPT1C, SOX8, caspase 9, ALOX12, IGF1R, SOD2, MYO5A, MBL2, PPARD, ERCC4, ERCC3, AURKA, MKI67, GLI1, DPYD, ABCC1
2011	Cibeira MT [74]	W	Multiple myeloma	28	Thalidomide-induced peripheral neuropathy	GSTT1
2011	Corthals SL [75]	W	Multiple myeloma	238	Bortezomib induced peripheral neuropathy	CYP17A1
2011	Favis R [76]	W	Myeloma	139	Bortezomib-induced peripheral neuropathy	CTLA4, PSMB1, CTSS, GJE1, DYNC1I1, TCF4
2011	Hong J [77]	A	Colorectal	52	Sensory neuropathy	GSTP1
2011	Johnson DC [78]	W	Multiple myeloma	1495	Thalidomide-related peripheral neuropathy	ABCA1, ICAM1, PPARD, SERPINB2, SLC12A6
2011	Leskela S [79]	W	Lung, breast, ovary, uterus, head and neck	118	Neurotoxicity	CYP2C8, CYP3A5
2011	Sucheston LE [80]	W, AA	Breast	888	Taxane-induced neurotoxicity	FANCD2
2012	Baldwin RM [81]	W, AA, A	Breast	855	Paclitaxel induced peripheral sensory neuropathy	FGD4, FZD3, EPHA5
2012	Braunagel D [82]	W	Acute myeloid leukemia	360	Cytarabine-induced neurotoxicity	NME1
2012	Fung C [83]	W, A, AA, H	Testicular germ cell tumor	137	Cisplatin-induced neurotoxicity, peripheral neuropathy	None
2012	Hasmats J [84]	W	Ovarian, lung, carcinoma in uteri/peritoneal/breast	94	Paclitaxel/carboplatin-induced neuropathy	ABCA1
2012	Hertz DL [85]	W, AA	Breast	111	Peripheral neuropathy	CYP2C8
2012	Leandro-Garcia LJ [86]	W	Ovary, lung, breast	214	Paclitaxel-induced peripheral neuropathy	TUBB2A
2012	Won HH [87]	A	Colon	96	Severe oxaliplatin-induced chronic peripheral neuropathy	TAC1, FOXC1, GMDS, ITGA1, PELO, ACYP2, TSPYL6, DLEU7, BTG4, POU2AF1, CAMK2N1, FARS2, LYRM4
2013	Argyriou AA [88]	W	Colorectal	200	Oxaliplatin-induced peripheral neuropathy	SCN4A, SCN10A
2013	Bergmann TK [89]	W	Ovarian	241	Paclitaxel induced neuropathy	None
2013	Cecchin E [90]	W	Colorectal	144	Oxaliplatin neurotoxicity	ABCC1, ABCC2
2013	de Graan AJ [91]	W	Esophagus, ovary, cervix, endometrial, breast, lung, head/neck	261	Paclitaxel-induced neurotoxicity	CYP3A4
2013	Hertz DL [92]	W, AA	Breast	209	Paclitaxel-induced neuropathy	CYP2C8
2013	Kumamoto K [93]	A	Colorectal	63	Oxaliplatin-induced sensory peripheral neuropathy	GSTP1, GSTM1
2013	Leandro-Garcia LJ [94]	W	Ovary, fallopian tube, peritoneum, lung, uterus, breast	144	Paclitaxel induced peripheral sensory neuropathy	EPHA4, EPHA6, EPHA5, XKR4, LIMK2
2013	Lee KH [95]	A	Colon	292	Sensory neuropathy	XRCC1
2013	Liu YP [96]	A	Gastric	126	Oxaliplatin-induced neurotoxicity	GSTP1
2013	McWhinney-Glass S [97]	N/A	Ovarian	404	Platinum/taxane-induced neurotoxicity	SOX10, BCL2, OPRM1, TRPV1
2013	Oguri T [98]	A	Colorectal	70	Oxaliplatin-induced chronic peripheral neurotoxicity	ACYP2, FARS2, ERCC1, TAC1
2014	Abraham JE [99]	W	Breast	1303	Taxane-related sensory neuropathy	ABCB1, TUBB2A, CYP2C8, ABCC2, CYP1B1, KIAA0146-PRKD, SLCO1B1, EPHA6
2014	Bhojwani D [100]	N/A	Acute lymphoblastic leukemia	369	Methotrexate-induced neurotoxicity	ASTN2, PXDC1, IYD
2014	Custodio A [101]	W	Colon	206	Oxaliplatin-induced peripheral neuropathy	CCNH, ABCG2
2014	Hertz DL [102]	W, AA, A	Breast	412	Paclitaxel-induced peripheral neuropathy	CYP2C8, ABCG1
2014	Khrunin AV [103]	W	Ovarian	104	Cisplatin-based neurotoxicity	None
2014	Lee SY [104]	A	Breast	85	Paclitaxel and gemcitabine combination chemotherapy neurotoxicity	RRM1

W: White; A: Asian; AA: African American; H: Hispanic

In Table 3, we summarize the focus genes from the literature review with respect to neuropathy induced by different chemotherapy agents, including platinum, taxane, platinum/taxane, Bortezomib, bortezomib/vincristine, thalidomide, methotrexate, cytarabine, platinum/fluorouracil, platinum/S-1 (i.e., oral fluoropyrimidine consists of tegafur, 5-chloro-2,4 dihydroxypyrimidine, and potassium oxonate), taxane/gemcitabine, platinum/fluorouracil/leucovorin, platinum/fluorouracil/irinotecan, prednisone/vincristine/methotrexate, platinum/capecitabine, platinum/fluorouracil/irinotecan/leucovorin and rituximab/cyclophosphamide/doxorubicin/vincristine/prednisone. Among the different (or combined) chemotherapy agents, those studied most frequently in relation to drug-induced neuropathy were platinum, taxane and the combination of platinum/taxane, for which our literature search respectively produced 21, 19 and 5 related papers.

**Table 3:** Summary of genes associated with chemotherapy agent-specified neuropathy from the literature review. Number of papers for each agent-specified neuropathy, number of genes associated with each agent-specified neuropathy and number of agent-specified neuropathies associated with each gene are shown. For the association between a gene and an agent-specified neuropathy, the number of relating papers is listed

	Agent	P	T	P/T	B	B/V	Th	M	Cyt	P/F	P/S	T/G	P/F/L	P/F/I	Pr/V/M	P/C	P/F/I/L	R/Cyc/D/V/Pr
Genes	# of papers	21	19	5	2	1	2	1	1	2	1	1	2	2	1	1	1	1
(IPA symbols)	# of genes	26	19	7	7	17	6	3	1	1	1	1	2	1	2	0	0	0
	# of agents																	
GSTP1	6	7	1							1	1		1	1				
ERCC1	2	2		1														
ACYP2	1	2																
FARS2	1	2																
GSTM1	1	2																
TAC1	1	2																
ABCC2	2	1	1															
ABCC1	2	1																
ABCG2	1	1																
AGXT	1	1																
BTG4	1	1																
CAMK2N1	1	1																
CCNH	1	1																
DLEU7	1	1																
FOXC1	1	1																
GMDS	1	1																
GSTM3	1	1																
ITGA1	1	1																
ITGB3	1	1																
KCNN3	1	1																
LYRM4	1	1																
PELO	1	1																
POU2AF1	1	1																
SCN10A	1	1																
SCN4A	1	1																
TSPYL6	1	1																
CYP2C8	2		5	1														
ABCB1	1		3															
EPHA5	1		2															
EPHA6	1		2															
TUBB2A	1		2															
CYP3A4	2		1												1			
CYP3A5	2		1												1			
ABCG1	1		1															
CYP1B1	1		1															
EPHA4	1		1															
FANCD2	1		1															
FGD4	1		1															
FZD3	1		1															
LIMK2	1		1															
SLCO1B1	1		1															
SPIDR	1		1															
XKR4	1		1															
ABCA1	2			1			1											
BCL2	1			1														
OPRM1	1			1														
SOX10	1			1														
TRPV1	1			1														
CTLA4	1																	
CTSS	1																	
CYP17A1	1																	
DYNC1I1	1																	
GJC3	1																	
PSMB1	1																	
TCF4	1																	
PPARD	2						1											
ALOX12	1					1												
AURKA	1					1												
CASP9	1					1												
CPT1C	1					1												
DPYD	1					1												
ERCC3	1					1												
ERCC4	1					1												
GLI1	1					1												
IGF1R	1					1												
MBL2	1					1												
MKI67	1					1												
MYO5A	1					1												
RHOBTB2	1					1												
SOD2	1					1												
SOX8	1					1												
GSTT1	1						1											
ICAM1	1						1											
SERPINB2	1						1											
SLC12A6	1						1											
ASTN2	1							1										
IYD	1							1										
PXDC1	1							1										
NME1	1								1									
RRM1	1											1						
XRCC1	1												1					

P: Platinum; T: Taxane; P/T: Platinum/Taxane; B: Bortezomib; B/V: Bortezomib/Vincristine; Th: Thalidomide; M: Methotrexate; Cyt: Cytarabine; P/F: Platinum/Fluorouracil; P/S: Platinum/S-1; T/G: Taxane/Gemcitabine; P/F/L: Platinum/Fluorouracil/Leucovorin; P/F/I: Platinum/Fluorouracil/Irinotecan; Pr/V/M: Prednisone/Vincristine/Methotrexate; P/C: Platinum/Capecitabine; P/F/I/L: Platinum/Fluorouracil/Irinotecan/Leucovorin; R/Cyc/D/V/Pr: Rituximab/Cyclophosphamide/Doxorubicin/Vincristine/Prednisone

Among the focus genes reported in the articles, *GSTP1*, *CYP2C8* and *ABCB1* were studied the most frequently (Table 4). *ABCC2* and *GSTP1* were associated with both platinum- and taxane-induced neuropathy; *CYP2C8* was associated with both taxane- and platinum/taxane-induced neuropathy; and *ERCC1* was associated with platinum- and platinum/taxane-induced neuropathy. Besides platinum-, taxane- and platinum/taxane- induced neuropathy, neuropathy induced by other chemotherapy agents were not frequently studied. Therefore, we focused on the genes associated with platinum-, taxane- and platinum/taxane-induced neuropathy in our analyses.

**Table 4:** Focus genes* associated with platinum-, taxane-, and platinum/taxane- induced neuropathy, as identified through the literature review

Platinum-induced neuropathy	Taxane-induced neuropathy	Platinum/Taxane-induced neuropathy
ABCC1	ABCB1	ABCA1
ABCC2	ABCC2	BCL2
ABCG2	ABCG1	CYP2C8
ACYP2	CYP1B1	ERCC1
AGXT	CYP2C8	OPRM1
BTG4	CYP3A4	SOX10
CAMK2N1	CYP3A5	TRPV1
CCNH	EPHA4	
DLEU7	EPHA5	
ERCC1	EPHA6	
FARS2	FANCD2	
FOXC1	FGD4	
GMDS	FZD3	
GSTM1	GSTP1	
GSTM3	LIMK2	
GSTP1	SLCO1B1	
ITGA1	SPIDR	
ITGB3	TUBB2A	
KCNN3	XKR4	
LYRM4		
PELO		
POU2AF1		
SCN10A		
SCN4A		
TAC1		
TSPYL6		

*Genes shown to be significant based on the literature

### IPA core analysis

**Fig. 1:**
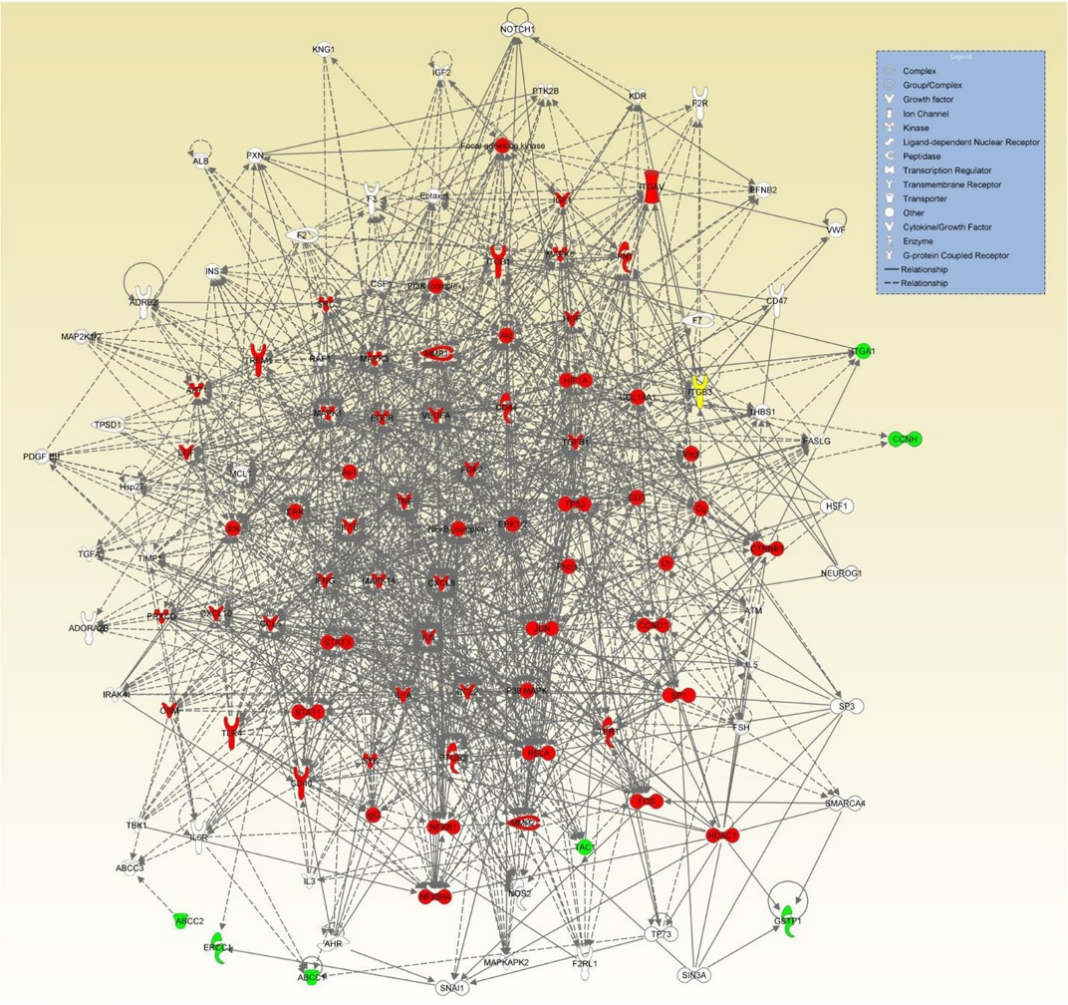
The most significant network (*p* value = 10^−12^) generated by IPA core analysis for 26 focus genes associated with platinum-induced neuropathy. Green: focus genes; red: genes with at least 15 connections; yellow: focus genes with at least 15 connections. Dashed and solid lines represent indirect and direct interactions, respectively

**Fig. 2:**
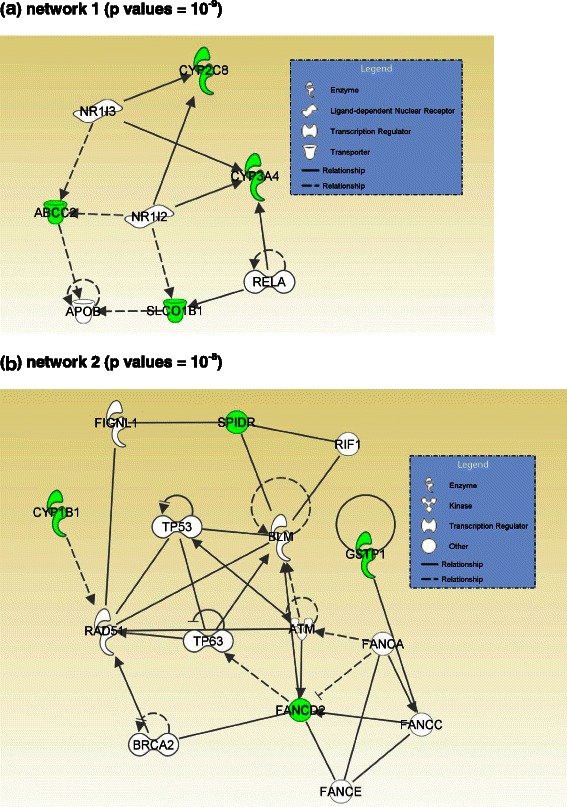
The most significant networks (*p* values = 10^−9^ and 10^−8^) generated by IPA core analysis for 19 focus genes associated with taxane-induced neuropathy. Green: focus genes. Dashed and solid lines represent indirect and direct interactions, respectively. **a** network 1 (*p* values = 10^−9^). **b** network 2 (*p* values = 10^−8^)

**Fig. 3:**
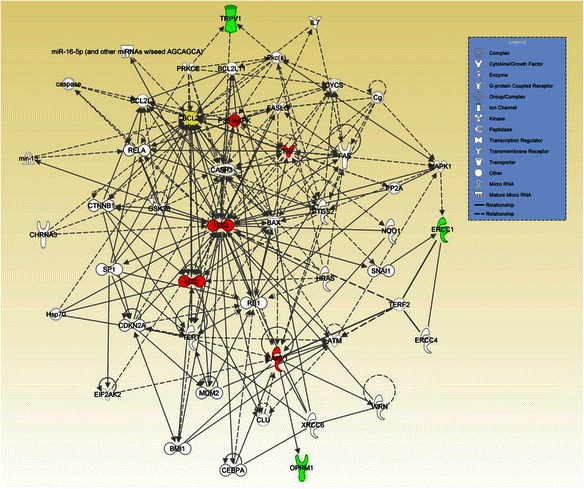
The most significant network (*p* value = 10^−8^) generated by IPA core analysis for 7 focus genes associated with platinum/taxane-induced neuropathy. Green: focus genes; red: genes with at least 15 connections; yellow: focus genes with at least 15 connections. Dashed and solid lines represent indirect and direct interactions, respectively

We performed the IPA core analysis for the focus genes reported to be associated with platinum-, taxane- and platinum/taxane- induced neuropathy. The significant networks revealed from the IPA core analyses are shown in Figs. 1, 2 and 3 for the focus genes reported to be associated with platinum-, taxane- and platinum/taxane- induced neuropathy, respectively. In the networks, the solid and dashed edges or arrows indicate direct and indirect interactions, respectively. In Table 5, we report the genes that had at least 15 connections (i.e., hubs, suggesting biological importance) in the networks, ranked by the number of connections for each gene.

**Table 5:** List of genes with at least 15 connections (i.e., hubs*) in the networks, ranked by the number of connections for each gene

Platinum-induced CIPN		Platinum/taxane-induced CIPN	
IPA Symbol	# of connections	IPA Symbol	# of connections
IL6	70	TP53	42
TNF	69	BCL2**	28
CXCL8	56	MYC	16
IL1B	55	PARP1	16
ERK1/2	54	P38 MAPK	15
VEGFA	52	TNF	15
MAPK1	51		
NFkB (complex)	46		
P38 MAPK	45		
TGFB1	43		
COL18A1	42		
CCL2	39		
IFNG	38		
PTGS2	37		
ERK	34		
TP53	34		
MAPK3	33		
Akt	32		
STAT3	30		
CD3	29		
JUN	29		
PI3K (complex)	29		
EGFR	28		
MMP1	28		
HGF	27		
Jnk	27		
CCL5	26		
CD40	26		
IL1A	26		
ITGB1**	26		
MMP2	25		
Cg	24		
FN1	24		
RELA	24		
TLR4	23		
Vegf	23		
CXCL10	22		
EGF	21		
ITGB3	21		
MAPK14	21		
NFKBIA	21		
SP1	21		
STAT1	21		
AKT1	20		
HIF1A	20		
SRC	20		
TERT	20		
Pkc(s)	19		
CTNNB1	18		
Focal adhesion kinase	18		
FOS	18		
HDAC1	18		
IgG	18		
ITGAV	18		
NFKB1	18		
CD44	17		
FGF2	17		
Lh	17		
MAPK8	17		
SYK	17		
Ap1	16		
CCND1	16		
IGF1	16		
PRKCD	16		
TREM1	16		
OSM	15		

*Suggests biological importance

**Focus genes

#### Platinum-induced neuropathy

The IPA core analysis revealed six networks associated with platinum-induced neuropathy. Using a nominal significance level of 10^−5^, of the 6 networks, we found only one network to be significant (*p* value of 10^−12^; Fig. 1. We note that 66 genes (one focus gene and 65 “novel” genes) out of 121 genes in the network have at least 15 connections (Table 5), suggesting the potential biological importance of these genes in CIPN associated with platinum-based chemotherapy. The gene *ITGB3* was the only focus gene in the network, and the top 5 “novel” genes were *IL6*, *TNF*, *CXCL8*, *IL1B* and *ERK1/2*.

#### Taxane-induced neuropathy

The IPA core analysis for taxane-induced neuropathy revealed eight networks, two of which were significant, with *p* values of 10^−9^ and 10^−8^ (Fig. 2). There is no hub in the network generated by the IPA core analysis of the focus genes reported to be associated with taxane-induced neuropathy.

#### Platinum/taxane-induced neuropathy

The IPA core analysis for platinum/taxane-induced neuropathy identified three networks, one of which was significant, with a *p* value of 10^−8^ (Fig. 3). We note that 6 genes (one focus gene and 5 additional “novel” genes) out of 48 genes in the network have at least 15 connections. The gene *BCL2* is the only focus gene included in the network that has more than 15 connections. The 5 additional genes that directly or indirectly interact with the corresponding focus genes associated with platinum/taxane-induced neuropathy based on the literature are *TP53*, *MYC*, *PARP1*, *P38 MAPK* and *TNF*.

## Discussion

In this study, we performed a comprehensive literature review to identify genes implicated in CIPN and then used IPA bioinformatic tools to conduct comprehensive pathway and network analyses of the known genes identified in the literature. Neurotoxicity is common in cancer patients who are treated with platinum compounds and anti-microtubule agents, and the development of CIPN is a potentially debilitating sequela. From the literature review, we found that neuropathy induced by platinum compounds and taxanes (and a combination of these two agents) has been studied most frequently. Neuropathy induced by chemotherapy agents other than platinum, taxane and platinum/taxane combinations has not been adequately studied.

Among the focus genes identified from our literature search, *GSTP1*, *CYP2C8* and *ABCB1* were most frequently assessed as candidates for CIPN. From the literature review, we also found that the genomic variations of genes associated with neuropathy induced by platinum versus taxane compounds were different. For example, *GSTP1*, *ERCC1*, *ACYP2*, *FARS2*, *GSTM1* and *TAC1* were found to be associated with platinum-induced neuropathy in more than one study but were not associated with taxane-induced neuropathy. On the other hand, *CYP2C8*, *ABCB1*, *EPHA5*, *EPHA6* and *TUBB2A* were found to be associated with taxane-induced neuropathy in more than one study, but not to be associated with platinum-induced neuropathy (Table 3). The overall theme is that these CIPN-associated genes are related to the networks that regulate intracellular drug concentrations (e.g., *GSTP1*, *GSTM1* and *ABCB1*), response to DNA damage (e.g., *ERCC1*, *FANCD2*, *BCL2*, and *SOX10*), cellular stress response pathways (e.g., *BCL2*), inflammation (e.g., *ABCC1*, *ABCC2*, *ABCG2*, *ITGA1*, *ITGB3*, *TAC1*, *ABCB1*, *ABCC2*, *EPHA4*, *EPHA6*, *SLCO1B1*, *TUBB2A*, *ABCA1*, *BCL2*, *OPRM1* and *TRPV1*), and neuronal plasticity (e.g., *ERCC1* and *TAC1*).

We performed IPA core analysis for the genes associated with platinum-, taxane- and platinum/taxane-induced neuropathy. We found that *IL6*, *TNF*, *CXCL8*, *IL1B* and *ERK1/2* were the top genes in terms of the number of connections in platinum-induced neuropathy, suggesting either direct or indirect interactions with nervous tissue leading to CIPN after exposure to platinum compounds. It is particularly interesting that studies of pain in cancer patients have shown the importance of cytokine genes [28–37] including *IL6*, *TNF* and *IL1B* polymorphisms. These studies hypothesized that cytokines associated with inflammation or tissue damage modify the activity of nociceptors, which contributes to pain hypersensitivity. Studies also suggest that hyperexcitability in pain transmission neurons may also be caused by proinflammatory cytokines produced by glial cells that respond to inflammation or other cancer-produced cytokines. Substance P and excitatory amino acids released from presynaptic terminals result to an exaggerated pain response [38, 39]. In patients with lung cancer, polymorphisms in *TNF* and *IL6* were significantly associated with pain severity (for *TNF*, GG = 4.12; GA = 5.38; AA = 5.50; *p* = 0.04) and with morphine-equivalent daily dose (*IL-6*, GG = 69.61; GC = 93.6; CC = 181.67; *p* = 0.004) [36]. An additive effect of mutant alleles in *IL1B T-31C* (odds ratio = 0.55, 95 % confidence interval = (0.31, 0.97)) was also found to be associated with high intensity of pain, depressed mood and fatigue in lung cancer patients [31].

In addition to the top connections in the networks, the overall biological processes involved in the networks help us to better understand the gene-phenotype association. The IPA core analysis is a process for creating molecule networks on the basis of focus genes, which are genes associated with the phenotypes of interest. Because all the focus and non-focus genes in the network have inter-connected relationships, it provides a list of novel candidate genes associated with the phenotype. The network also provides a clearer picture of the (possibly interacting) genes that might be directly or indirectly associated with chemotherapy-induced peripheral neuropathy. The most significant network generated by IPA core analysis for the focus genes associated with platinum-induced neuropathy (Fig. 1) contains genes for inflammation (multiple interleukins, *TNF*, *IFNG*, *STAT3*, *STAT1*), DNA damage response (*TP53*) and cell survival (*MAPK*, *JUN*, *ERK*, *NFkB*). Network 2, which relates to taxane-induced neuropathy (Fig. 2b), includes many genes that are involved in the DNA damage response. The network related to neuropathy induced by combined platinum and taxane therapy (Fig. 3) resembles Fig. 1 in terms of the cellular functions involved, i.e., inflammation, DNA damage response and cell survival. The major commonality among Figs. 1, 2b and 3 is *TP53*, which is a central hub in these three networks. Network 1, which relates to taxane-induced neuropathy (Fig. 2a), primarily involves drug metabolizing enzymes and transporter proteins that will affect the intracellular concentration of taxanes. These analyses suggest that genetic variations in the DNA damage response are associated with the risk of developing CIPN, and that taxane-induced neuropathy is also affected by genetic variations that regulate intracellular drug levels while this aspect may not be important for platinum compounds.

This bioinformatic approach to expanding gene networks and identifying connection hubs has limitations. First, many proteins do not interact, while others may connect to major hubs that interact with hundreds of genes and proteins. Therefore, it is believed that the degree of connectivity obeys a power law, which means that the network is scale-free, a desired property. However, we found that the IPA metric/algorithm that generates networks does not guarantee that the resulting networks are scale-free, even though the networks may exhibit certain scale-free behavior in which the major hubs are closely followed by smaller ones that have less connectivity, and the smaller hubs are then followed by other nodes with an even smaller degree of connectivity, and so on (see Figs. 1 and 3). Furthermore, the IPA algorithm that generates networks will not continue if the network reaches the pre-specified maximum network size (i.e., 140 genes), which might rule out many nodes with small degrees of connectivity and impact the scale-free behavior. We employed a widely used log-log plot to investigate whether the networks in Figs. 1 and 3 follow the power law [24, 40]. The log-log plot should appear as a decaying straight line if the network obeys the power law, which was not observed in our plot. Therefore, we cannot conclude that the resulting networks are scale-free.

Further limitations could be that the connections may be specific to certain tissues or physiological contexts that are not applicable to CIPN. Many of the connections have not been demonstrated in neural tissue. Nevertheless, this network analysis identified biological processes that are relevant to the mechanism of neuropathy induced by platinum compounds and taxanes, thus providing the basis for future studies of the genes involved in these biological processes. Our study has not discovered any pathways involved in pain perception. Perhaps, due to the fact that many studies in the literature were done as focused search for SNP associations in a relatively small set of genes in pre-selected pathways, such as glutathione, DNA repair, cell cycle, apoptosis, cell signaling, and metabolism. Whether new gene sequencing technology can discover genetic markers associated with differences in neuropathic pain perception remains to be seen. In conclusion, our study has shown putative genes associated with CIPN. Future studies will include the selection of pharmacogenomic panel tests that will help identify patients at risk for CIPN and the routine incorporation of such panels into clinical practice.
